# The relationship between osteoclastogenic and anti-osteoclastogenic pro-inflammatory cytokines differs in human osteoporotic and osteoarthritic bone tissues

**DOI:** 10.1186/1423-0127-19-28

**Published:** 2012-03-01

**Authors:** Janja Zupan, Radko Komadina, Janja Marc

**Affiliations:** 1 University of Ljubljana, Faculty of Pharmacy, Department of Clinical Biochemistry, Askerceva cesta 7, SI-1000 Ljubljana, Slovenia; 2 General Hospital Celje, Department of Traumatology, Oblakova 5, SI-3000 Celje, Slovenia

**Keywords:** Interleukins, Interferons, TNF-α, TGF-β1, β_3 _integrin, Cathepsin K, OSCAR

## Abstract

**Background:**

Pro-inflammatory cytokines possess osteoclastogenic or anti-osteoclastogenic activities. They influence osteoclasts directly or via the receptor activator of nuclear factor κB (RANK), RANK ligand (RANKL) and osteoprotegerin (OPG) system. Recent evidence suggests that inflammation may play a role in osteoporosis (OP) and osteoarthritis (OA). We aimed therefore to determine whether there is a difference between both groups: first, in the expression of the osteoclastogenic and anti-osteoclastogenic cytokines, second, in correlation of these cytokines with bone mineral density (BMD) and levels of bone turnover markers (BTM) and third, in correlation between the expression of these cytokines and osteoclast specific genes and RANK/RANKL/OPG genes.

**Methods:**

Human bone samples from 54 age and sex matched patients with OP or OA were collected during hip arthroplasty surgery. The expression of 25 genes encoding pro-inflammatory cytokines, their receptors, osteoclast specific genes and RANK/RANKL/OPG genes was measured using quantitative real-time PCR. Total hip, femoral neck and lumbar spine BMD and BTM in blood samples were measured. The comparison between OP and OA was assessed using Student's *t*-test or Mann-Whitney *U *test and correlations between gene expression, BMD and BTM were determined using nonparametric correlation.

**Results:**

The results demonstrated a higher expression of interleukin (IL)-6 and IL-1α in OP, and interferon (IFN)-γ in OA (*p *< 0.0005). Negative correlations of total hip BMD with tumor necrosis factor-α (TNF-α) in OA and with RANKL/RANK in OP were found (*p *< 0.05). Significant correlations with BTM were shown for IL-1α and IFN-γ in OP (rho = 0.608 and -0.634) and for TNF-α, IL-6 and transforming growth factor-β1 (TGF-β1) in OA (rho = 0.591, -0.521 and 0.636). Results showed OP specific negative correlations (IFN-γ with *ITGB3*, IFN-β1 with *CTSK*, tartrate resistant acid phosphatase (TRAP), *CALCR*, RANK, RANKL, IL-1α with *CTSK*, OPG, IL-17A with *CALCR*) and positive (TGF-β1 with *CTSK*, TRAP, RANK), and OA specific negative (IL-1α with osteoclast associated immunoglobulin-like receptor (*OSCAR*), TNF-α with RANK, RANKL, OPG) and positive (IL-6 with RANK, RANKL, OPG) correlations.

**Conclusions:**

Our results demonstrate that the relationship between osteoclastogenic and anti-osteoclastogenic pro-inflammatory cytokines differs in human OP and OA bone and could present an important factor for characteristics of OP and OA bone phenotypes.

## Background

Osteoclasts are influenced by a variety of pro-inflammatory osteoclastogenic and anti-osteoclastogenic cytokines that can either stimulate or suppress their activity [1]. This regulation of osteoclasts becomes particularly important in the pathological activation of the immune system, when pro-inflammatory cytokines are produced extensively by activated T cells [2]. As the immune system is also triggered during estrogen deficiency or inflammation, both osteoporosis (OP) and osteoarthritis (OA) are being recently considered as inflammation driven bone disorders [3,4]. Increased levels of IL-1, TNF-α and IL-6 after the menopause have been associated with OP [5-8] and higher incidence of non-traumatic fractures was associated with higher levels of serum IL-6 [9]. Furthermore, serum IL-6 accounted for up to 34% of the total variance of change in bone mineral density (BMD) after the menopause [10]. Blockade of TNF and IL-1 reduced bone resorption in postmenopausal OP women [11].

Anti-osteoclastogenic cytokines such as IFN-γ and IFN-β have been shown *in vitro *to strongly suppress osteoclastogenesis by inhibiting receptor activator of nuclear factor κB (RANK) signalling [12,13]. However, under conditions of inflammation and estrogen deficiency, this effect of IFN-γ could be overpowered by T cell secretion of RANKL and TNF-α, resulting in net bone loss [2,14]. Transforming growth factor β1 (TGF-β1) cannot be classified as a pro-inflammatory cytokine, however its role in maintaining a balance, by mediating both inhibition and stimulation of bone resorption and formation, could be deregulated by the pro-inflammatory cytokines released in pathological conditions of bone [15].

Pro-inflammatory cytokines, such as IL-1α, TNF-α and IL-17 exhibit osteoclastogenic properties [16,17] with many synergistic and also antagonizing interactions between them [18-21] and some of them, such as IL-6, may produce both stimulating and suppressing actions on osteoclasts [17,22,23].

*In vitro *studies have shown that these cytokines can influence osteoclasts directly via their specific receptors located on osteoclasts or via modulation of the RANK/RANK ligand (RANKL)/osteoprotegerin (OPG) system [24-26]. IL-1α, IL-6 and TNF-α can act on osteoclasts directly or by the RANK/RANKL/OPG pathway [16,22,27]. Regardless of the pathway, the activation of osteoclasts leads to the expression of the osteoclast specific genes *CALCR, ITGB3, OSCAR, CTSK *and *ACP5 *that encode calcitonin receptor, β_3 _integrin, osteoclast associated immunoglobulin-like receptor (OSCAR), cathepsin K and tartrate resistant acid phosphatase (TRAP) respectively, all of which are involved in differentiation, activation and survival of osteoclasts [28].

These data, obtained mainly from *in vitro *studies and experimental animal models, indicate complex crosstalk between the pro-inflammatory cytokines, and strongly suggest that the exact outcome of the specific cytokine must be evaluated in pathological conditions of the bone microenvironment. One of the first studies on human bone biopsies, performed by Ralston in 1994, demonstrated that the expression of IL-1α, IL-1β, TNF-α and IL-6 is more frequent in women with OP fractures than in normal postmenopausal women [29]. Recently, D'Amelio et al. found up regulation of expression of RANKL and OPG genes in OP, while TGF-β was highly expressed in OA women [30]. To the best of our knowledge the expression of anti-osteoclastogenic cytokines, such as IFN-γ and IFN-β, has not been quantified in human OP or OA bone tissue so far. Due to ethical reasons, normal human bone tissue is difficult to obtain and studies utilizing normal human iliac crest bone [29] and intertrochanteric bone obtained from cadavers at autopsy [31,32] are limited.

OP and OA are two contrasting bone phenotypes in terms of BMD [33], and both have only recently been considered as inflammatory bone disorders [3,4] in which osteoclastogenic and anti-osteoclastogenic cytokines might play important roles.

Given these observations the aim of our study was to investigate, whether there is a difference between OP and OA: first, in the expression of the osteoclastogenic and anti-osteoclastogenic cytokines, second, in correlation of these cytokines with BMD and levels of bone turnover markers (BTM) and third, in correlation of these cytokines with osteoclast specific genes and RANK/RANKL/OPG genes.

Therefore, the gene expression of 15 pro-inflammatory cytokines including their receptors, TGF-β1 and its receptor, 3 genes of the RANK/RANKL/OPG system and 5 osteoclast specific genes, in human OP and OA bone tissue, was examined. IFN-γ and IFN-β have been quantified for the first time in human OP and OA bone tissue.

## Methods

### Human bone tissue samples

Gene expression profiles were determined in bone samples from fifty four (54) patients undergoing hemi-arthroplasty or total hip arthroplasty because of low-energy femoral neck fracture (23 OP patients) or primary hip OA (31 OA patients). Patients were included in the study in a consecutive manner over a period of 1.5 years as they were directed to arthroplasty at the Department of Traumatology in the General Hospital Celje because of diagnosis of OP or OA. OP was diagnosed by radiologically confirmed low-energy femoral neck fracture and diagnosis of OA was established by clinical and radiographic criteria according to the Harris hip score [34]. All OP patients were submitted to arthroplasty within 24 h following femoral neck fracture. Bone tissue samples (approximately 1 cm^3^) were collected during surgical procedures of femoral osteotomy from the trabecular bone at the metaphyseal cutting plane. Bone samples were immediately frozen in liquid nitrogen and stored at -80°C until RNA extraction. The exclusion criteria for enrollment of OP and OA patients, verified by the questionnaire, laboratory results and interview, included the following: secondary OP or OA, liver and kidney diseases, endocrinological disorders and medical anamnesis on receiving medications with known influence on bone metabolism. The study was approved by the ethical committee of Republic of Slovenia and all patients gave written informed consent.

### Bone mineral density measurement

BMD at the contralateral hip, lumbar spine (L2-L4) and femoral neck was measured by dual-energy X-ray absorptiometry (Hologic QDR 1000, Hologic, Inc. Bedford). The measurement of BMD in OA patients was performed pre-operatively and in OP patients immediately post-operatively.

### Biochemical markers of bone turnover

Biochemical BTM were measured in a subset of 28 patients (12 OP and 16 OA) within 24 days after surgery. Blood samples were collected between 8:00 a.m. and 10:00 a.m. after an overnight fast. Serum C-terminal crosslinking telopeptides of type I collagen (CTX), serum free soluble RANKL, cathepsin K and OPG were measured by enzyme immunoassay (Serum CrossLaps ELISA, Nordic Bioscience Diagnostics A/S, Herlev, Denmark; sRANKL ELISA, Cathepsin K ELISA and Osteoprotegerin ELISA, Biomedica, Vienna, Austria) with an inter-assay coefficient of variation (CV) below 10%, 6%, 8% and 8%, respectively. Osteocalcin (OC) in heparinized plasma was measured by a solidphase, two-site chemiluminescent enzyme-labelled immunometric assay (Immulite Osteocalcin, Diagnostic Product Corporation, Los Angeles, CA, USA).

### Quantitative real-time polymerase chain reaction

Total RNA was extracted from human bone samples and complementary DNA (cDNA) synthesized according to our previously described procedure [35] and stored at -80°C until measurement of gene expression.

Predesigned and validated gene-specific TaqMan Gene Expression Assays (Applied Biosystems, Foster, CA, USA) for RANK (*TNFRSF11A*), IFN-γ (*IFNG*), *IL1A*, IL-1α receptor type I (*IL1R1*), IL-6 (*IL6*), TNF-α receptor (*TNFRSF1A*) and *OSCAR *genes (Hs00921374_m1, Hs99999041_m1, Hs00899848_m1, Hs00168392_m1, Hs00174131_m1, Hs01042313_m1 and Hs01100185, respectively) were used according to the manufacturer's protocol. For the remaining genes, oligonucleotides (Sigma Aldrich Chemie Gmbh, Munich, Germany) for mRNA encoding IFN-γ receptor (*IFNGR1*), IFN-β (*IFNB1*), both chains of IFN-β receptor (*IFNAR1 *and *IFNAR2*), IL-1α receptor type II (*IL1R2*), IL-6 receptor (*IL6R*), IL-17A (*IL17A*), two IL-17A receptors A and C (*IL17RA *and *IL17RC*), TNF-α (*TNF*), TGF-β1 (*TGFB1*), TGF-β1 receptor TβR1 (*TGFBR1*), calcitonin receptor (*CALCR*) and β_3 _integrin (*ITGB3*) were designed using Primer-BLAST (NCBI). Real time quantification was carried out on LightCycler 480 (Roche Diagnostics Ltd, Rotkreuz, Switzerland) using 5× HOT FIREPol EvaGreen qPCR Mix Plus for oligonucleotides and 5× HOT FIREPol Probe qPCR Mix Plus (Solis BioDyne, Tartu, Estonia) for TaqMan assays. All samples were quantified in triplicate. Dilution series of cDNA were prepared to create a relative standard curve with each run and absolute quantification of the data was performed using the second derivative maximum method (LightCycler 480, Software Version 1.5, Roche Diagnostics Ltd, Rotkreuz, Switzerland). All data were normalized to the geometric mean of two internal housekeeping genes, glyceraldehyde-3-phosphate dehydrogenase (*GAPDH*) and ribosomal protein, large, P0 (*RPLP0*). Data on *TNFSF11, TNFRSF11B, ACP5 *and *CTSK *expression were taken from our previous study on the same group of patients [35] and the appropriate correction factor, according to expression of two housekeeping genes, was applied.

### Statistical analysis

Variables were tested for normality of distribution using the Shaphiro-Wilk test. For variables such as age, sex, body mass index and BMD, that have met the normality assumption, Student's *t*-test was used for comparison between the two groups of patients. Because of the nonparametric distribution of the BTM levels and mRNA data, Mann-Whitney *U *test was used for comparison between the two tissue groups, and Spearman rho correlation analysis for estimating the relationship between the gene expression data, BMD and BTM, and within the mRNA data, respectively. Results with a *p *value of 0.05 or less were considered statistically significant. All data analyses were performed using PASW software, version 18 (IBM, Chicago, IL, USA).

## Results

### Study population

The study population consisted of age and sex matched patients with femoral neck fracture due to low-energy trauma (OP) and patients with arthroplastic surgery of the hip (OA). The two groups differed in body mass index (BMI) and BMD values of the hip, femoral neck and lumbar spine, these values being significantly lower in OP than in OA. Cathepsin K and OPG serum levels were significantly higher in OP (Table 1).

**Table 1:** Anthropometric characteristics and serum levels of bone turnover markers

	Osteoporosis	Osteoarthritis
**Age (years)**	74.1 ± 7.7	71.8 ± 4.2

**Sex (women/men)**	17/6	23/8

**Body mass index (kg/m^2^)**	24.5 ± 2.3	28.3 ± 4.4**

**Hip bone mineral density (g/cm^2^)**	0.726 ± 0.135	0.880 ± 0.140**

**Femoral neck BMD (g/cm^2^)**	0.615 ± 0.098	0.757 ± 0.131**

**Lumbar spine L2-L4 BMD (g/cm^2^)**	0.861 ± 0.184	0.994 ± 0.193*

**CTX (pmol/l)**	3078(1285;5391)	3547(2183;4967)

**Osteocalcin (μg/l)**	6.80(0.30;11.98)	7.90(1.03;17.03)

**Cathepsin K (pmol/l)**	12.0(10.0;22.1)	9.4(6.7;11.3)*

**RANKL (pmol/l)**	0.01(0.01;0.02)	0.01(0.01;0.06)

**OPG (pmol/l)**	5.86(4.52;7.47)	4.42(4.09;5.01)*

*BMD *bone mineral density. *CTX *C-terminal crosslinking telopeptides of type I collagen. *RANKL *receptor activator of nuclear factor κB ligand.

*OPG *osteoprotegerin.

Values are means ± standard deviation (with the exception of sex) and medians(25th;75th quartile) for bone turnover markers.

Comparisons were assessed with Student's t-test or Mann-Whitney *U *test, *p < 0.05, **p < 0.001.

### Gene expression measurement

The expression of 25 genes encoding 15 pro-inflammatory cytokines and their receptors (*IL1A, IL6, IFNG, IFNB1, IL17A, TNF, IL1R1, IL1R2, IL6R, IFNG1, IFNAR1, IFNAR2, IL17RA, IL17RC, TNFRSF1A*), TGF-β1 and its receptor (*TGFB, TGFBR1*), 3 genes of the RANK/RANKL/OPG system (*TNFRSF11A, TNFSF11, TNFRSF11B*) and 5 osteoclast specific genes (*ITGB3, OSCAR, CTSK, ACP5, CALCR*) in human OP and OA bone tissue was measured. All mRNA values were normalized to the geometric mean of *GAPDH *and *RPLP0 *mRNA. Ligand to receptor mRNA levels for each of the studied cytokine ligand receptor pairs were calculated from normalized expression data and these ratios were used further in our correlation analyses. Excluding Background, the name of the specific pro-inflammatory cytokine is referred to the corresponding ligand receptor mRNA ratio throughout the manuscript.

### Difference in gene expression between OP and OA

The results of Mann-Whitney U tests showed no differences between males and females in the whole study group or within OP or OA groups in the expression levels of genes measured.

**Figure 1:**
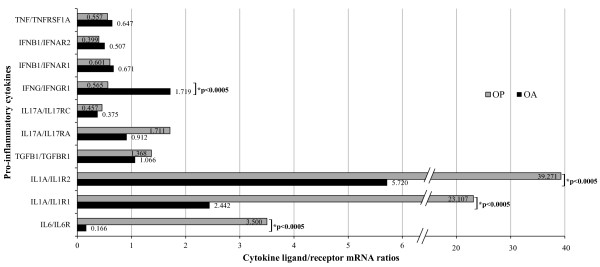
**Quantitative real-time PCR data of the pro-inflammatory cytokine mRNA levels in osteoporosis (OP) and osteoarthritis (OA)**. Ligand to receptor ratios for each of the studied cytokine ligand receptor pairs were calculated from mRNA values normalized to geometric mean of *GAPDH *and *RPLP0 *mRNA. Values are medians. Comparisons were assessed by the Mann-Whitney *U *test, *p values < 0.05 were considered statistically significant.

The expression of osteoclastogenic and of anti-osteoclastogenic cytokines between OP and OA tissues were compared using the Mann-Whitney *U *test. In OP tissue, a significantly higher expression of ligand to receptor mRNA levels for *IL1A*/*IL1R1, IL1A*/*IL1R2 *and *IL6/IL6R *were observed, while in OA tissue, there was a significantly higher expression of IFN-γ ligand to receptor mRNA (Figure 1). Looking at the osteoclast specific genes, there was higher expression of *OSCAR *and *CALCR *in OA, while higher expression of *ACP5 *in OP was of limited statistical significance (*p *= 0.049). *TNFRSF11A *and *TNFSF11 *were both significantly higher in OP, while their ratio did not reach the level of significance. *TNFRSF11B *was similar between both groups, while the ratio with RANKL (*TNFSF11/TNFRSFS11B*) was significantly higher in OP (Table 2).

**Table 2:** Data on mRNA levels of osteoclast specific genes and RANK/RANKL/OPG genes in OP and OA

Gene symbol	Gene name	OP (n = 23)	OA (n = 31)	OP/OA^a^	*p *value^b^
**Osteoclast specific genes**					

***OSCAR***	Osteoclast associated immunoglobulin-like receptor	0.574(0.345;0.854)	1.002(0.732;1.339)	0.54	0.002*

***ITGB3***	β_3 _integrin	0.241(0.074;0.586)	0.285(0.143;0.994)	0.85	0.231

***CTSK***	Cathepsin K	0.288(0.121;1.109)	0.263(0.136;0.555)	1.10	0.786

***ACP5***	Tartrate resistant acid phosphatase (TRAP)	0.270(.128;1.011)	0.147(0.073;0.259)	1.84	0.049*

***CALCR***	Calcitonin receptor	0.223(0.138;0.644)	0.860(0.482;1.562)	0.26	< 0.0005**

**RANK/RANKL/OPG genes**					

***TNFRSF11A***	Receptor activator of nuclear factor κB (RANK)	1.580(0.687;3.939)	0.573(0.355;1.038)	2.76	0.002*

***TNFSF11***	Receptor activator of nuclear factor κB ligand (RANKL)	0.528(0.076;1.038)	0.091(0.027;0.253)	5.80	< 0.0005**

***TNFRSF11B***	Osteoprotegerin (OPG)	0.699(0.410;0.914)	0.776(0.308;1.615)	0.90	0.681

***TNFSF11/TNFRSF11A***	RANKL/RANK	0.241(0.083;0.928)	0.140(0.064;0.271)	1.72	0.076

***TNFSF11/TNFRSF11B***	RANKL/OPG	0.448(0.295;1.601)	0.112(0.075;0.193)	4.00	< 0.0005**

Values are medians(25th;75th percentile). All mRNA values were normalized to the geometric mean of *GAPDH *and *RPLP0 *mRNA.

^a^Gene expression ratio of medians in osteoporotic (OP) versus osteoarthritic (OA) patients.

^b^Comparisons were assessed by the Mann-Whitney *U *test. *p < 0.05, **p < 0.001.

### Correlation of the pro-inflammatory cytokines with bone mineral density

The correlation between cytokine ligand to receptor mRNA levels and BMD was determined by nonparametric analysis. In OP, significant negative association with both, hip and femoral neck BMD was found for RANKL/RANK (rho = -0.452 and -0.443, *p *< 0.05). In OA, significant negative correlation was observed for *TNF*/*TNFRSF1A *with hip BMD (rho = -0.390, *p *< 0.05), while correlation with femoral neck BMD did not reach the level of significance (rho = -0.355, *p *= 0.058).

Significant negative correlations of RANKL/OPG with total hip and femoral neck BMD (*p *= -0.306 and -0.383, *p *< 0.05) were found in the whole study group only.

### Correlation of the pro-inflammatory cytokines with bone turnover markers

The correlation between cytokine ligand to receptor mRNA levels and BTM was determined by nonparametric analysis. In OP, significant positive association for *IL1A*/*IL1R2 *with serum RANKL and negative for IFN-γ ratio with serum cathepsin K have been found (rho = 0.608 and -0.634, *p *< 0.05). In OA, significant positive correlation was observed for TGF-β1 ratio with serum OPG and TNF-α ratio with cathepsin K (rho = 0.636 and 0.591, *p *< 0.05), and a negative correlation of IL-6 ratio with cathepsin K (rho = -0.521, *p *< 0.05). Correlations between RANKL, RANKL/RANK, RANKL/OPG mRNA and BTM were not significant for any of the groups studied (*p *> 0.05), while OPG mRNA showed significant negative correlation with serum OPG in the OA group (rho = -0.518, *p *< 0.05).

### Correlation of the pro-inflammatory cytokines with osteoclast specific and RANK/RANKL/OPG genes

The correlation between cytokine ligand to receptor mRNA levels and osteoclast specific genes, RANK, RANKL and OPG gene mRNA was examined by nonparametric analysis (Table 3).

**Table 3:** Correlation of the expression of the pro-inflammatory cytokines and osteoclasts' specific genes and RANK/RANKL/OPG genes

Gene mRNA	OSCAR		β3 integrin		Cathepsin K		TRAP		Calcitonin receptor		RANK		RANKL		OPG	
**Ligand/Receptor mRNA ratio**	**OP**	**OA**	**OP**	**OA**	**OP**	**OA**	**OP**	**OA**	**OP**	**OA**	**OP**	**OA**	**OP**	**OA**	**OP**	**OA**

***IL6/IL6R***	-0.282	-0.215	0.587*	-0.363*	-0.451*	0.519**	-0.329	0.319	-0.431*	0.656**	-0.421	0.658**	0.083	0.619**	-0.147	0.529**

***IL1A/IL1R1***	-0.181	-0.156	0.239	0.137	-0.508*	-0.166	-0.350	0.021	-0.494*	-0.434*	-0.311	-0.301	-0.259	-0.258	-0.484*	-0.296

***IL1A/IL1R2***	-0.021	-0.380*	0.100	-0.005	-0.103	0.325	0.015	0.348	-0.084	0.207	0.027	0.305	-0.066	0.337	-0.491*	0.271

***TGFB1/TGFBR1***	0.054	-0.198	0.112	0.256	0.473*	-0.075	0.486*	0.048	0.302	-0.201	0.531*	-0.051	0.263	-0.208	0.027	-0.033

***IL17A/IL17RA***	0.007	0.030	-0.116	0.167	-0.491*	-0.081	-0.263	-0.084	-0.600**	0.087	-0.605**	0.002	-0.293	-0.009	0.068	0.021

***IL17A/IL17RC***	-0.140	0.010	-0.358	-0.070	-0.390	-0.154	0.213	-0.004	-0.600*	-0.095	-0.537*	-0.096	-0.005	-0.027	0.213	-0.004

***IFNG/IFNGR1***	0.198	-0.181	-0.601**	-0.062	0.119	0.205	-0.018	0.097	0.191	0.120	-0.070	0.207	-0.197	0.296	0.337	0.167

***IFNB1/IFNAR1***	0.224	0.006	0.095	0.188	-0.553**	-0.283	-0.453*	-0.290	-0.527**	-0.038	-0.580**	-0.202	-0.447*	-0.186	0.002	-0.070

***IFNB1/IFNAR2***	0.313	-0.049	0.013	0.107	-0.510*	-0.137	-0.430*	-0.152	-0.463*	0.064	-0.517*	-0.052	-0.456*	-0.035	0.043	0.063

***TNF/TNFRSF1A***	0.329	0.292	-0.038	0.018	-0.428*	-0.632**	-0.429*	-0.598**	-0.414*	-0.613**	-0.362	-0.631**	-0.356	-0.541**	0.192	-0.534**

*OSCAR *osteoclast associated immunoglobulin-like receptor. *TRAP *tartrate resistant acid phosphatase. *RANK *receptor activator of nuclear factor κB. *RANKL *RANK ligand. *OPG *osteoprotegerin.

The numbers are Spearman's rho coefficients for osteoporotic (OP) and osteoarthritic (OA) tissue. *p *values were obtained using nonparametric bivariate correlation analysis, *p < 0.05, **p < 0.01. Significant positive correlations are underlined with bold line.

In OP specifically, IL-6 showed a significant positive correlation with *ITGB3 *and TGF-β1 with *CTSK *and *ACP5*. Significant negative correlations were found for the IL-6 with *CTSK *and *CALCR, IL1A*/*IL1R1 *ratio with *CTSK*, and IL-17A with *CALCR *for ligand to receptor ratios with both of its receptors IL-17RA and IL-17RC, while *IL17A*/*IL17RA *also showed negative correlation with *CTSK*. Significant negative correlation of both *IL1A*/*IL1R1 *and *IL1A*/*IL1R2 *with OPG was found. Only TGF-β1 showed significant positive correlation with RANK. Negative correlations were demonstrated for IL-17A ligand to receptor ratios, with both IL-17RA and IL-17RC, and for IL-6 ratio with RANK.

Anti-osteoclastogenic cytokines in OP tissue showed a significant negative correlation for IFN-γ ligand to receptor ratio (*IFNG*/*IFNGR1*) with *ITGB3 *and for IFN-β ratio with *CTSK, CALCR *and *ACP5*. The latter observations were found for both ligand to receptor mRNA ratios, *IFNB1*/*IFNAR1 *and *IFNB1*/*IFNAR2*, as there are two protein chains of IFN-β receptor. A significant negative correlation of both *IFNB1*/*IFNAR1 *and *IFNB1*/*IFNAR2 *with RANK and RANKL gene expression was also found.

In OA tissue, interestingly, correlations directly opposite to those in OP tissue were found for IL-6 ratio with *ITGB3, CTSK *and *CALCR*. Furthermore, the ratio of IL-1α to its receptor type II (*IL1A*/*IL1R2*) showed a negative correlation with *OSCAR*. Strong negative correlation of TNF-α ratio with RANK, RANKL and OPG, and an opposite, positive correlation of IL-6 ratio with the same genes, were found.

No significant correlations for IFN-γ and IFN-β were found with any of the osteoclast specific genes in OA.

In both tissues, *IL1A*/*IL1R1 *was significantly inversely correlated with *CALCR*, while TNF-α ratio showed significant negative correlations with *CTSK, ACP5 *and *CALCR*.

## Discussion

We have demonstrated that the relationship between osteoclastogenic and anti-osteoclastogenic pro-inflammatory cytokines differs in human OP and OA bone.

The comparison of OP and OA showed higher expression of osteoclastogenic cytokines IL-6 and IL-1α in OP and higher expression of IFN-γ in OA. Negative association with BMD has been found for RANKL in OP and for TNF-α in OA. In OP, positive association of IL-1α with serum RANKL and negative of IFN-γ with serum cathepsin K have been found, while in OA, positive correlation of TGF-β1 with serum OPG and of TNF-α with cathepsin K, and negative correlation of IL-6 with cathepsin K were observed. The correlations of IL-6 with osteoclasts specific genes *ITGB3, CTSK *and *CALCR *expression were directly opposite between OP and OA. The negative correlations specific to OP were IL-1α with *CTSK *and OPG, IL-17A with *CALCR*, IFN-γ with *ITGB3*, IFN-β with *CTSK, ACP5, CALCR*, RANK and RANKL and positive correlations specific to OP were TGF-β1 with RANK, TRAP and cathepsin K. The negative correlations specific for OA were IL-1α with *OSCAR *and TNF-α with RANK, RANKL, OPG, and positive OA specific IL-6 with RANK, RANKL and OPG.

OP and OA are both age related skeletal disorders, in which the involvement of pro-inflammatory cytokines, due to pathological activation of the immune system, was recently suggested [3,4]. As OP and OA are two opposite bone phenotypes in terms of BMD [33], our hypothesis was that the two types of tissue differ in the relationship between osteoclastogenic and anti-osteoclastogenic pro-inflammatory cytokines and in the correlation of these pro-inflammatory cytokines with BMD and BTM. Bone samples were collected from OP and OA patients with significantly different BMD at three skeletal sites (Table 1). The results show higher expression of IL-6 and IL-1α in OP, while the expression of IFN-γ was higher in OA tissue. Although our results were significant only for IL-1α, IL-6 and IFN-γ, higher expression of all osteoclastogenic cytokines studied, except TNF-α was implied in OP, and higher expression of the anti-osteoclastogenic cytokines IFN-γ and IFN-β in OA (Figure 1). The higher expression of genes encoding bone resorption molecules TRAP, RANK and RANKL and RANKL/OPG in OP tissue confirms the adequacy of the system used in our study. In OA, *OSCAR *and *CALCR *were significantly higher expressed (Table 2). Interestingly, a previous study comparing OA with controls from cadavers found no differences in *CALCR *expression [32].

Our results show that the relationship between osteoclastogenic cytokines IL-6 and IL-1α and the anti-osteoclastogenic cytokine IFN-γ is diametrically opposite in OP and OA, corresponding to their bone phenotype. In accordance with *in vitro *studies, higher level of the anti-osteoclastogenic cytokine IFN-γ in OA might contribute to the suppressed osteoclast activity [13], while higher levels of the osteoclastogenic cytokines IL-6 and IL-1α in OP could suggest enhanced osteoclastogenesis, leading to increased bone loss [1,16,20,22]. Our results of higher expression of IL-6 and IL-1α in OP and no difference in TNF-α expression are in accordance with those of Ralston [29]. Furthermore, our results for IL-6 also coincide with those of Cauley et al. who showed that high serum levels of IL-6 predict a higher incidence of non-traumatic fractures. They found similar association for TNF-α, while in our study TNF-α ratio showed negative correlation with total hip BMD in OA [9]. Nevertheless, the negative correlations of RANKL/RANK in OP and RANKL/OPG in the whole group with total hip and femoral neck BMD found in our study are in accordance with the well-established role of the RANK/RANKL system in bone [24]. We have found tissue specific correlation of the pro-inflammatory cytokines gene expression and serum levels of BTM such as cathepsin K, RANKL and OPG. In OP, positive association of IL-1α with serum RANKL might indicate the osteoclastogenic activity of IL-1α, while negative association of IFN-γ with serum cathepsin K might indicate anti-osteoclastogenic activity of IFN-γ, previously confirmed *in vitro *in mice [36]. In OA, positive correlation for TGF-β1 with serum OPG and a negative correlation of IL-6 with cathepsin K, that could indicate the anti-osteoclastogenic actions of these cytokines, resulting in bone sparing effect in OA, were found. The latter observation for IL-6 is in accordance with the previously observed dual role of IL-6 on osteoclasts [17,22,23]. Although previous reports have shown an inverse relationship between RANKL bone mRNA and serum levels in OA males [37], our results of this correlation analysis did not reach the level of significance. The reason is most likely due to low statistical power as there were only three OA males with serum RANKL levels. Interestingly, we observed significant negative correlation between OPG mRNA and serum OPG in the OA group although Findlay et al. have found the opposite correlation.

Our results of non-significant higher expression of TGF-β1 ligand gene *TGFB1 *in OA (*p *= 0.053, data not shown) and higher expression of RANKL/OPG in OP (*p *< 0.0005) correspond to results of the recent D'Amelio et al. study, that they have also verified on the protein level [30]. To the best of our knowledge, the expression of IFN-γ and IFN-β has not been previously quantified in human OP or OA bone tissue.

Moreover, we performed a correlation analysis of the pro-inflammatory cytokines and TGF-β1 expression with osteoclast specific and RANK/RANKL/OPG genes. Again, the differences between OP and OA were found. IL-6 showed positive correlation with β_3 _integrin expression in OP, while a negative link with the same gene has been found in OA. Furthermore, negative correlation of IL-6 expression with genes encoding RANK, cathepsin K and calcitonin receptor in OP and the positive correlation with the same genes in OA, have been found. The results for IL-6 in OA are in accordance with the previously observed opposing data on IL-6 activity on osteoclasts [17,22,23]. Nevertheless, the 21-fold higher expression of IL-6 in OP and its positive correlation with β_3 _integrin expression could indicate its osteoclastogenic effect in OP. Furthermore, both IL-6 and IFN-γ have shown directly opposite correlation with β_3 _integrin expression between OP and OA, and also reverse correlation between them (rho = -0.463, *p *< 0.05, data not shown) that was specific for OP.

Another osteoclastogenic cytokine in our study, IL-1α has shown positive correlation with serum RANKL and negative correlation with OPG gene expression in OP that might indicate its osteoclastogenic pathway. The negative association of IL-1α with cathepsin K gene expression is contrary to the results of Kamolmatyakul et al. in mice. Negative correlations of IL-1α with OSCAR gene expression specific to OA could indicate the dual role of IL-1α, similar to that of IL-6. Nevertheless, IL-1α showed almost 10-fold higher expression in OP than in OA. Our results might suggest that IL-1α in OP could act via suppression of the OPG, the decoy receptor for RANKL, resulting in less inhibition of RANKL, increased serum RANKL and enhanced osteoclast activity observed in OP. Additional studies are needed to confirm this suggestion.

The differences between OP and OA were also found for TGF-β1, a ubiquitous growth factor retaining a balance in coupling bone resorption and formation [15]. TGF-β1 showed positive correlation with cathepsin K, TRAP and RANK gene expression in OP that could suggests the involvement of TGF-β1 in enhanced osteoclastogenesis in OP. Similarly, Yan et al. showed that TGF-β1 stimulates the expression of *CALCR *and RANK in TRAP positive osteoclasts in isolated murine monocytic cells [38]. On the other hand, TGF-β1 positive correlation with serum OPG in OA indicates its opposite, anti-osteoclastogenic effect in OA. Furthermore, our results showed a negative correlation of TGF-β1 with IFN-β in OP tissue (rho = -0.536, *p *< 0.05, data not shown), that is in accordance with the mechanism proposed by Lovibond et al., in which TGF-β1 stimulates osteoclast formation via suppression of the anti-osteoclastogenic effect of IFN-β [19].

Looking at the anti-osteoclastogenic cytokines in our study, OP specific negative correlations of IFN-γ with β_3 _integrin and of IFN-β with genes encoding cathepsin K, calcitonin receptor, TRAP, RANK and RANKL were found, that are in accordance with the anti-osteoclastogenic effect of IFN-γ and IFN-β, proved *in vitro *[12,13]. These relationships were specific for OP, although we would have expected such connections indicating the bone sparing effect in OA. However, in our study, IFN-γ was 3-fold higher expressed in OA associated with high bone mass. Previously, IFN-γ has been shown to down regulate cathepsin K expression in co-cultures of mouse osteoclasts and stromal cells [36], while in our study, negative correlation of IFN-γ with β_3 _integrin gene was found that has not been described before and should be further examined. Taking together the results for IL-6 and IFN-γ, i.e. their inverse relationship, their inverse link to their common target β_3 _integrin gene, and their inverse expression between OP and OA, we suggest that the difference in relationship between osteoclastogenic cytokine IL-6 and anti-osteoclastogenic cytokine IFN-γ could contribute to different osteoclast activity and thus might present an important factor for the distinctive bone phenotypes seen in OP and OA.

For the last two cytokines TNF-α and IL-17A no difference between OP and OA was observed, moreover the negative correlations with osteoclast specific and RANK/RANKL/OPG genes are contrary to the previous reports on their osteoclastogenic activity [16,17,21,25]. Negative association of TNF-α with BMD, RANK, RANKL and OPG gene expression and a positive with serum cathepsin K (rho = 0.591, *p *< 0.05), might also indicate the dual role of TNF-α on osteoclastogenesis. However, further studies are needed.

To summarize, our results of 1) higher expression of IL-6 and IL-1α in OP, OP specific negative correlation of RANKL with BMD, positive of IL-1α with serum RANKL and negative correlation of IFN-γ with serum cathepsin K, and a positive link of IL-6 with β_3 _integrin expression and negative of IL-1α with OPG 2) TGF-β1 positive correlation with cathepsin K, TRAP and RANK gene expression in OP and TGF-β1 positive correlation with serum OPG in OA and 3) higher expression of IFN-γ in OA, IFN-γ negative link to β_3 _integrin expression, and IFN-β negative correlation with cathepsin K, calcitonin receptor, TRAP, RANK and RANKL gene expression in OP, demonstrate the difference in the relationship between osteoclastogenic and anti-osteoclastogenic pro-inflammatory cytokines in OP and OA bone.

The limitations of the current study present a relatively low number of patients with BTM and the lack of further functional confirmation of the significant associations found in our study. As blood samples in this study were collected after surgery, it might be argued there could have been influence of surgery or immobilization on levels of BTM. To test this hypothesis, initial comparison of BTM levels in blood samples collected within 7 days after surgery with blood samples collected within 8-24 days after surgery was performed, and the results showed no influence of the time of blood specimen collection on the levels of BTM studied. Due to difficulty in obtaining normal human bone tissue, no controls such as cadaveric bone samples [31,32], were included in our study. However, the augmentation of the pro-inflammatory cytokines is characteristic of the pathological conditions of bone, such as inflammatory, age and hormone related bone disorders. The advantage of our study is that we evaluated the human bone tissue *ex vivo *as it is by comparing the steady-state of the pro-inflammatory cytokine gene expression in bone tissue, originating from bone and bone marrow cells, between the two opposite bone phenotypes. To avoid possible post-fracture response affecting the steady-state of gene expression, samples were taken from intertrochanteric region, located distal to the site of fracture. The significant correlations found in our study suggest the pathways for further evaluation of the pro-inflammatory cytokines in OP and OA.

## Conclusions

In conclusion, we have found that the relationship between osteoclastogenic and anti-osteoclastogenic pro-inflammatory cytokines differs in human OP and OA bone tissue, corresponding to their specific bone phenotypes. Our data support the recent reports on the role of inflammation in OP and OA, and provide new pathways for the further investigation on the role of the pro-inflammatory cytokines in human OP and OA.

## Abbreviations

OP: Osteoporosis; OA: Osteoarthritis; BMD: Bone mineral density; BTM: Bone turnover markers; RANK: Receptor activator of nuclear factor κB; RANKL: RANK ligand; OPG: Osteoprotegerin; IL-: Interleukin-; IFN-: Interferon-; TNF-α: Tumor necrosis factor-α; TGF-β1: Transforming growth factor β1; *CALCR*: Calcitonin receptor gene; *ITGB3*: β_3 _integrin gene; OSCAR: Osteoclast associated immunoglobulin-like receptor; *CTSK: *Cathepsin K gene; TRAP: Tartrate resistant acid phosphatase; *ACP5: *Tartrate resistant acid phosphatase gene.

## Competing interests

The authors declare that they have no competing interests.

## Authors' contributions

JZ conceived of the study, performed the gene expression analysis, analysed the data and drafted the manuscript; RK organized the collecting of the human bone and blood samples and the patients' data, JM supervised the whole study from the beginning to the accomplished manuscript preparation. All authors read and approved the final manuscript.
